# Parasites, Bacteria, and Associated Pathological Changes in the Digestive System of Diurnal and Nocturnal Raptors in Central Italy

**DOI:** 10.3390/pathogens10121567

**Published:** 2021-11-30

**Authors:** Giacomo Rossi, Giuliana Terracciano, Riccardo Gherardi, Livio Galosi, Stefania Perrucci

**Affiliations:** 1 School of Biosciences and Veterinary Medicine, University of Camerino, 62024 Matelica, Italy; giacomo.rossi@unicam.it (G.R.); livio.galosi@unicam.it (L.G.); 2 Istituto Zooprofilattico Sperimentale delle Regioni Lazio e Toscana, 56123 Pisa, Italy; giuliana.terracciano@izslt.it; 3 Department of Veterinary Sciences, University of Pisa, 56124 Pisa, Italy; ric_gherardi@hotmail.com

**Keywords:** helminths, protozoa, bacteria, digestive system, pathological lesions, raptors, central Italy

## Abstract

The knowledge of raptor pathogens and associated lesions may be extremely important to enhancing raptor conservation efforts and reducing pathogen spillover to humans and domesticated animals and vice versa. Parasite infections of the digestive system and associated bacteria and pathological changes were evaluated in deceased diurnal and nocturnal raptors in central Italy. Overall, the prevalence of parasites (nematodes, cestodes, trematodes, acanthocephalans, and protozoa) identified in the examined birds was 72.41%, and most of the positive raptors (71.42%) showed multiple parasite infections. Among bacteria, *Salmonella typhimurium*, *Salmonella enterica* subspecies *diarizonae*, *Escherichia coli*, *Clostridium perfringens*, *Yersinia enterocolitica,* and *Pasteurella multocida* were identified. The results obtained showed that both parasites and bacteria may cause severe lesions in the digestive system of diurnal and nocturnal raptors; parasites and bacteria may concur in causing these lesions; most severe lesions are caused by the interaction of multiple pathogens, both parasites and bacteria; and the same pathogen taxa are frequently associated with the same pathological changes. This study is the first report of *S. typhimurium* and *S. enterica* subspecies *diarizonae* in *Buteo buteo*, while *Andracantha mergi*, *Spirocerca* spp., *Sarcocystis dispersa,* *Sarcocystis columbae,* and *Eumonospora* spp. were recorded for the first time in Italy.

## 1. Introduction

Raptors play a fundamental role in ecosystems as apex predators and scavengers and are considered biological indicators of environmental pollution. Therefore, studies on pathogens of raptors may provide useful data for ecosystem health monitoring, the evaluation of the health status of raptors populations, and the role these birds may play in spreading some important pathogens, such as drug-resistant bacteria and potentially zoonotic bacteria and parasites [1,2,3,4]. These animals are protected species in many countries, such as in Italy [5]. Nonetheless, worldwide, many raptor species are highly threatened [6]. In Italy, some diurnal and nocturnal raptors are currently included among endangered species, such as the marsh harrier (*Circus aeruginosus*), the Eleonora’s falcon (*Falco eleonorae*), the Egyptian vulture (*Neophron percnopterus*), and the Eurasian pygmy owl (*Glaucidium passerinum*) [6].

Environmental deterioration and modifications due to human activities, in combination with other factors, such as direct persecution, are the main causes that are currently contributing to the decline of raptor populations [4,7,8,9,10,11]. The increase in urbanized areas and the conversion of natural environments to crop and livestock production with the destruction of wooded areas have greatly reduced habitats suitable for nesting, migratory stops, and hunting of raptors [9,11].

Furthermore, land reclamation interventions with the reduction in humid areas and the modernization of agricultural activities have caused the removal of hedges, trees, and old ruins that may play an important role in causing a decrease in the diversity of prey animals hunted by these birds and of refuge and rest places for both prey and predators [9,11].

Pollution and pesticides and other drugs used in agricultural and zootechnical activities and ingestion of lead with the ingestion of shot prey may be responsible for acute or chronic poisoning of raptors, causing the death of these birds or negatively affecting their reproduction and ability to overcome diseases [12].

Moreover, many people and hunters still consider these birds as harmful to game animals, a disturbance for hunting from stationary stalking, or these birds are the subject of superstitions and popular beliefs, especially nocturnal raptors [1,13,14]. This is proved by the high number of shot and poisoned raptors recovered in wild animal centers during the hunting season each year [15].

Human activities may also contribute to the global dissemination of pathogens, thereby threatening wildlife [2,16]. Indeed, raptors are susceptible to several bacterial pathogens of humans and domestic animals [2] that are now considered contributing factors to the progressive decline of raptor populations [17,18].

Although most parasitic infections appear to cause little or no distress to healthy individuals, parasites may be the cause of severe health issues when they occur in high numbers or when they are associated with other concurrent diseases or stressing factors [19,20]. Nevertheless, some protozoa and helminth species may affect raptor flying performance [21,22] and predatory effectiveness [23], as well as they may predispose raptors to secondary trauma [24]. Moreover, lesions caused by endoparasite species are often complicated by secondary bacterial infection [25]. However, some bacteria, such as *Pasteurella multocida*, *Chlamydia,* and *Campylobacter* species, are frequently reported as primary causes of infections and/or diseases in these birds [11,17,26,27,28,29].

Reports on the occurrence and pathological changes associated with parasites and bacteria in raptors are scarce [18,30,31,32]. Nevertheless, these data may be extremely useful to improve the knowledge on the impact these pathogens may have on raptor health and to evaluate whether their pathogenic role is linked to parasite infection intensity, to a specific parasite or bacterial species, and to the concurrence of parasites and bacteria [20,25,30,31,33]. Furthermore, wild birds may act as potential vectors or reservoirs of pathogens for domestic animals and are suspected sources for human bacterial infections [2,17,27]. Therefore, the knowledge of raptor pathogens may be extremely important to enhance bird conservation efforts and reduce pathogen spillover to other animals and humans [27].

In this study, protozoan and helminth infections of the digestive system and associated bacteria and pathological lesions were evaluated in deceased raptors in central Italy.

## 2. Results and Discussion

Most of the birds examined in this study were admitted to wildlife recovery centers and died from trauma, often caused by gunshot (Table 1). Nevertheless, catarrhal and/or hemorrhagic (gastro) enteritis and cachexia were frequently observed in raptors showing or not traumatic lesions (Table 1).

Overall, the prevalence (72.41%) of parasites found in the digestive system of examined birds was high, as 21 out of 29 examined raptors scored positive for at least a parasite species. In most positive birds (14/21, 66.67%), the positivity for nematodes and protozoa was evidenced also at coprological analysis. Moreover, most of the positive raptors (15/21, 71.42%) showed multiple parasite infections (Table 2), as frequently observed in previous European studies [24,30,33,34,35,36,37,38].

Among identified parasites, helminths and especially nematodes (20/21, 95.23%) were more frequently recorded, as all positive raptors scored positive for these parasites, except for a barn owl found infected only by protozoa (Table 2). Capillariid nematodes were found in almost all the raptor species here examined and in most of the positive birds (15/21, 71.42%), followed by *Procyrnea* spp. (10/21, 47.61%), even if these latter nematodes were more frequently found among diurnal raptors (Table 2). Trematodes and, mainly, cestodes were more rarely found (Table 2).

Acanthocephalans were also frequently identified, especially in diurnal raptors (Table 2), while *Sarcocystis* spp. and *Eumonospora* spp. protozoa were identified in a few birds (Table 2).

Most of the pathogens identified in this study are commonly found in European diurnal and nocturnal raptors [8,23,24,30,35,37,38,39,40,41,42,43,44,45,46].

Lesions of different typologies and levels of severity were evidenced at histopathological analysis in the different digestive tracts of the examined raptors (Table 3).

Moreover, among selected bacteria, secondary bacterial infection and bacteria considered primary causes of infections and/or diseases in these birds were identified at bacteriological analysis (Table 4) [11,17,26,27,28,29].

**Figure 1:**
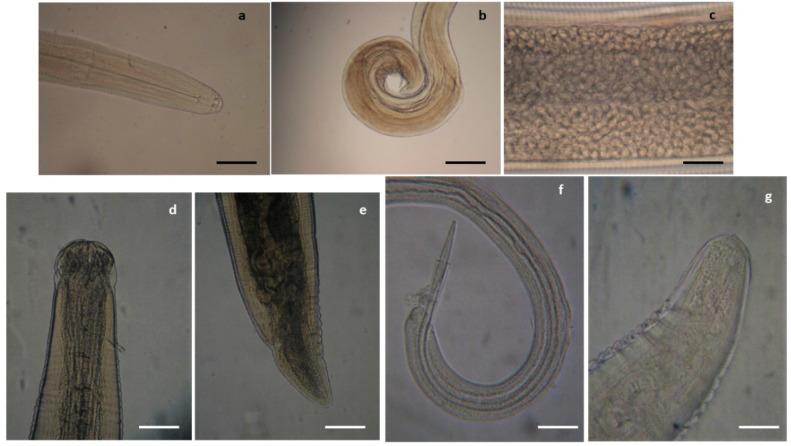
Nematodes identified in the common buzzard. (**a**) Anterior end of *Procyrnea mansioni* adult female, (**b**) caudal end of *P. mansioni* adult male, and (**c**) detail of a section of the body of a *P. mansioni* female showing a high number of eggs, scale bar 4 mm (**a,b**), 8 mm (**c**). Anterior (**d**) and caudal (**e**) end of *Porrocoecum angusticolle* adult female, scale bar 150 µm. (**f**) Caudal end of *Baruscapillaria falconis* adult male, scale bar 100 µm. Detail of the caudal end of *Physaloptera alata* (**g**), scale bar 600 µm.

**Figure 2:**
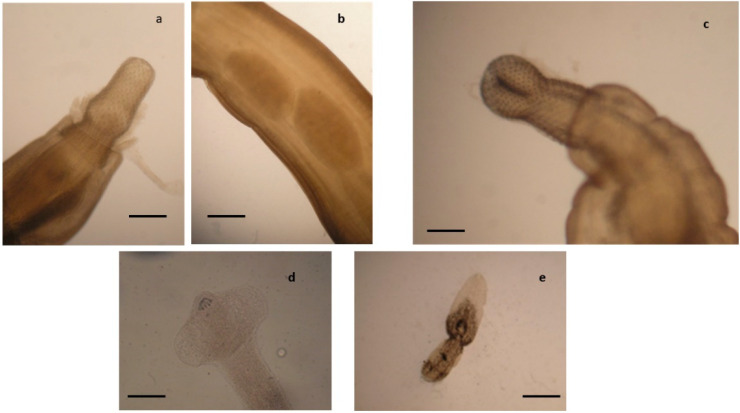
Acanthocephalans, cestodes, and trematodes identified in the European common buzzard (*B. buteo*). Anterior end (**a**) of *Centrorhynchus globocaudatus* showing the proboscis and detail of the body showing the two testicles (**b**), scale bar 0.6 mm. Anterior end (**c**) of *Centrorhynchus aluconis*, scale bar 0.6 mm. Anterior end of *Cladotaenia globifera* (**d**), scale bar 95 µm. (**e**) *Neodiplostomum attenuatum* €, scale bar 0.5 mm.

Among examined diurnal raptor species, all buzzards were found infected by *Procyrnea mansioni* in the gizzard and proventriculus, *Porrocoecum angusticolle* and *Baruscapillaria falconis* in the small intestine and *Centrorhynchus* spp. in the large intestine, although in some cases, the infection intensity was very different among the examined buzzards (Table 2, Figure 1 and Figure 2). Moreover, *Eucoleus dispar* in the esophagus of the common buzzards 3 and 4, *Physaloptera alata* in the gizzard of the common buzzard 1, *Neodiplostomum attenuatum* and *Spirocerca* spp. in the intestine of the common buzzard 2, and *Cladotaenia globifera* cestodes in the intestine of the common buzzard 4, were also identified (Table 2, Figure 1 and Figure 2).

Regarding bacteriological analysis, *Salmonella* *typhimurium* and *Salmonella enterica* subspecies *diarizonae* were detected in the liver and intestine of the common buzzard 2 and 3, respectively (Table 4). To the best of our knowledge, this study is the first report of *S. typhimurium* and *S. enterica* subspecies *diarizonae* in the common buzzard (*B. buteo*).

Gross lesions were found mainly in the digestive tract of the common buzzards 2, 3, and 4 (Table 1).

**Figure 3:**
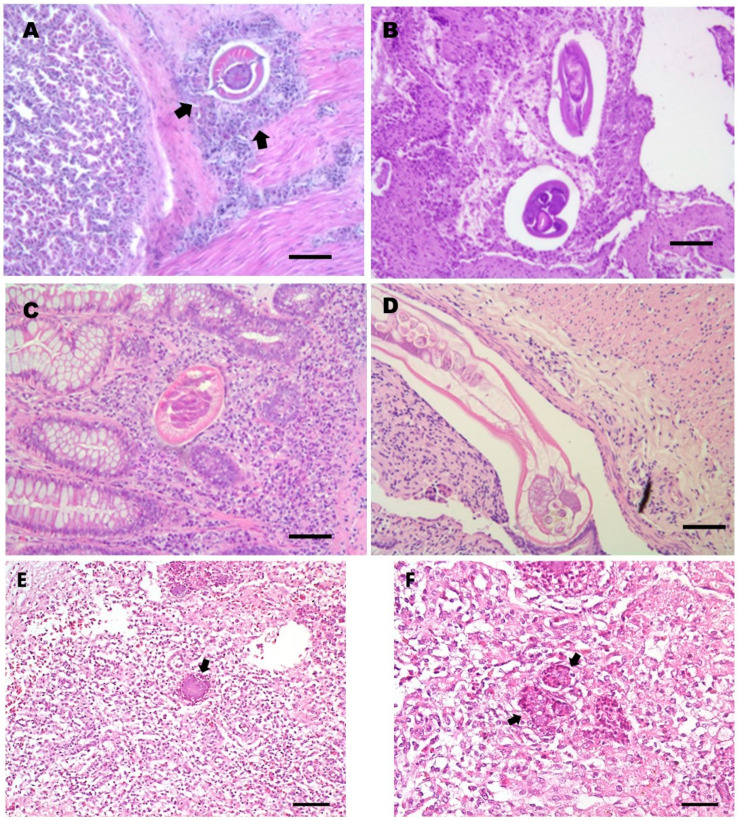
(**A**) Common buzzard (*B. buteo*), histology of the gizzard infected by *P. mansioni*, at the level of the isthmic area. Granuloma with the presence of a parasite; the granuloma wall consists of a mixed population of mono- and polymorph nuclear cells (heterophilic granulocytes, numerous lymphocytes, plasma cells, and macrophages—arrows). The lesion is recently formed and not well encapsulated by peri-nodular fibroblasts proliferation. (**B**) Common buzzard (*B. buteo*) histology of the proventriculus infected by *P. mansioni*: two parasites inside the mucosa can be observed, surrounded by a pyogranulomatous inflammatory infiltrate. Note the necrotic areas around the two parasites and the ulceration of the proventricular mucosa above the area of mucosal inflammation. (**C**) Common buzzard (*B. buteo*) histology of the small intestine with severe parasite colonization (mainly by *B. falconis*). The histological section shows a parasite localized in a deep portion of the mucosa in the small intestinal crypt area, surrounded by an acute inflammatory reaction, characterized by a large number of heterophils and eosinophils granulocytes, in the absence of mesenchymal reaction and/or fibrosis. (**D**) Honey buzzard (*Pernis apivorus*); histology of the jejunum parasitized by *Raillietina apivori*, characterized by chronic-active inflammation, infiltrating a segmental tract of the organ wall, with the presence of a parasite in the lumen. (**E**,**F**) Common buzzard (*B. buteo*), histology of the liver in a *Salmonella typhimurium*-infected bird: note the classic micro-granulomas, also called “typhoid-like” nodules, which show a center of unstructured necrosis surrounded by some inflammatory cells represented by heterophilic granulocytes and some mononuclear cells (arrows) without a capsule. H&E; scale bar = 500 µm (A, B, and E); 250 µm (C, D, and F).

At the histopathological examination, areas of mucosal erosion, as well as hyperemia, foci of micro- and macro-granulomatosis, and chronic-active inflammation were evidenced in the gizzard and in the proventriculus of all these buzzards and especially in the isthmus in two of them (Table 3, Figure 3a,b). These lesions were found constantly observed in *P.* *mansioni*-infected birds, and the number and severity of these lesions were also found related to *P. mansioni* intensity, which was higher in two buzzards (Table 2). In previous studies [30,45], dark red nodular lesions, erosions, and ulcers of the gastric mucosa associated with *Procyrnea* sp. infection have been reported in diurnal raptors, including buzzards, while data on associated histological lesions are lacking.

Inflammation of the intestinal mucosa and infiltration by heterophils and macrophages and micro and macro-granulomatous lesions were also found constantly present in the large intestine of *Centrorhynchus* spp.-infected buzzards. These findings confirm previous observations in buzzards, and some authors consider these lesions a direct consequence of the mechanical damage caused by the proboscis these parasites use to anchor themselves to the intestinal wall [30,37].

As previously reported [30,37,46], esophageal inflammation, characterized by chronic and diffuse inflammation with transmural infiltrate consisting of lymphocytes and plasma cells, was highlighted in the esophagus of the two *E. dispar*-infected common buzzards.

In the small intestine of a buzzard found heavily infected by *B. falconis*, foci of acute inflammation characterized by microscopic agglomerates composed by heterophils and eosinophils and focal mucosal erosion were highlighted (Figure 3C). Previous data on *B. falconis*-associated lesions are lacking. However, similar lesions have been observed in raptors infected by other intestinal capillariid species [47,48].

In a common buzzard, diffuse duodenal inflammation with the presence of transmural infiltrates of lymphocytes and plasma cells was found associated with a heavy infection caused by the trematode *N. attenuatum,* confirming previous findings [30,37], while mucosal erosion and severe hyperemia were evidenced in the small intestine of a buzzard found infected by a high number of *C. globifera* cestodes (Table 3).

Moderate lesions consisting of atypical and micro-granulomatous lesions sub-miliaric in size characterized by foci of caseous necrosis surrounded by some epithelioid cells or 2–3 macrophages, lymphocytes, and heterophilic granulocytes, in the absence of an encapsulating reactive fibrous wall (“*typhoid-like*”nodules), were only observed at histopathological examination (Figure 3E,F) in the two *Salmonella*-infected buzzards (Table 3). These findings agree with those generally found in *Salmonella*-infected birds [49]. *Salmonella* spp. infections are infrequently reported in raptors [1,46]. However, *Salmonella* spp. have been detected in the feces of a variety of asymptomatic raptors, especially those living in rescue centers for wild animals [26,50,51,52,53,54,55,56,57]. Raptors can also acquire the infection by preying on infected animals [17,28].

**Figure 4:**
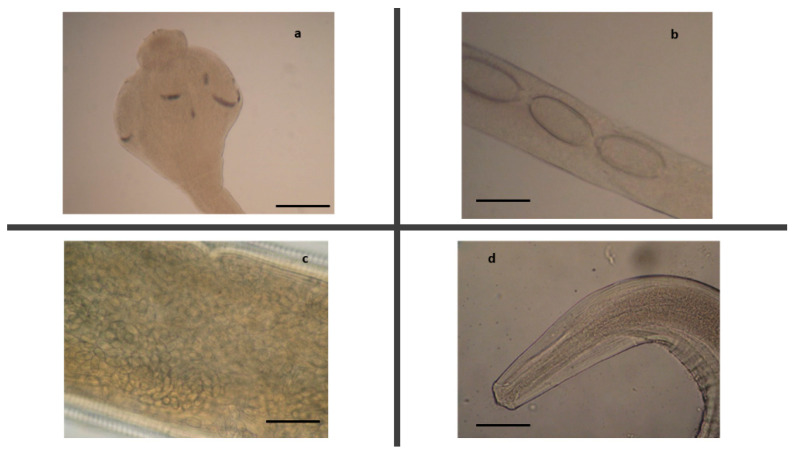
Parasites identified in the honey buzzard. (**a**) Scolex of *Raillietina apivori*, scale bar 200 µm. (**b**) Detail of the body of a *Baruscapillaria falconis* adult female with eggs, scale bar 50 µm. (**c**) Detail of the body of a *Procyrnea leptoptera* adult female with eggs, scale bar 2 mm. (**d**) Anterior end of *Physaloptera apivori*, scale bar 0.4 mm.

Among the two positive honey buzzards, *E. dispar*, *Physaloptera apivori*, and *Raillietina apivori* were identified, respectively, in the esophagus, gizzard, and intestine (Figure 3D) of honey buzzard 1 concurring with intestinal *S. typhimurium* infection, while honey buzzard 2 scored positive for *E. dispar* in the esophagus, *Procyrnea leptoptera* in the stomach, and *B. falconis* in the small intestine, along with *E. coli* infection in the liver (and brain) (Table 2 and Table 4, Figure 4). However, macroscopic lesions of the digestive system associated with severe weight loss and dehydration were observed mainly in the honey buzzard 2. This was also evidenced at the histopathological examination, as the presence of helminths in the esophagus and gizzard of the honey buzzard 1 was found not associated with specific lesions (Table 3), while chronic-active inflammation was found associated with *R. apivori* (Figure 3D). Conversely, granulomatous lesions were observed in the esophagus and proventriculus of the honey buzzard 2, associated with *E. dispar* and *P. leptoptera* infection, respectively. Interestingly, lesions found associated with *P. leptoptera* infection in this honey buzzard are very similar to those found in some of *P. mansioni*-infected European common buzzards examined here.

Furthermore, the presence of atypical sub-miliaric micro-granulomatous lesions was observed at the histopathological examination in the *S. typhimurium*-infected honey buzzard 1, while chronic and diffuse inflammation with transmural infiltrates made of lymphocytes and plasma cells were highlighted throughout the intestinal tract of honey buzzard 2. These latter lesions, along with hepatomegaly and nephromegaly observed at necropsy, could be associated mainly with *E. coli* infection. In fact, *E. coli* is a bacterial pathogen often isolated from the intestinal content of diurnal birds of prey, both as a primary pathogen or a pathogen secondary to other infections [2,18], and may cause acute septicemic forms or sub-acute-chronic infections characterized by voluminous granulomas, aero-sacculitis, pneumonia, pericarditis, osteomyelitis, and nephritis [17,46]. Clinically, diarrhea, anorexia, and respiratory distress can be also observed [2,18,58].

**Figure 5:**
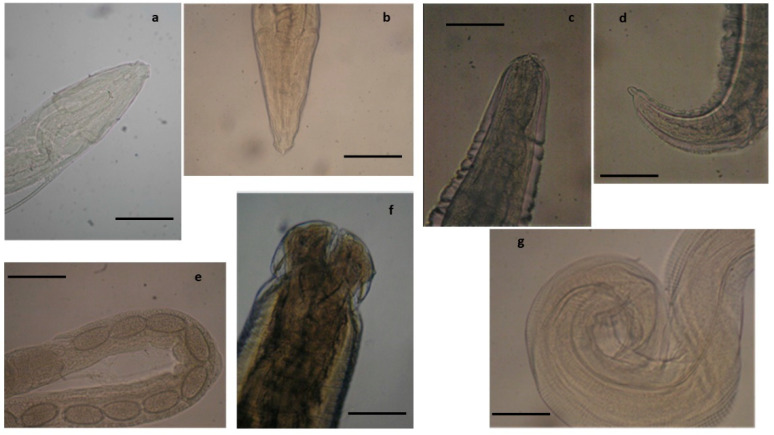
Nematodes identified in the sparrowhawk. Anterior (**a**) and posterior (**b**) end of *Synhimantus* (*Dispharynx*) *falconis*, scale bar 300 µm. Anterior (**c**) and posterior (**d**) end of *Physaloptera alata*, scale bar 500 µm. (**e**) Detail of a capillariid adult female with eggs, scale bar 70 µm. (**f**) anterior end of *Porrocoecum angusticolle*, scale bar 0.5 mm. (**g**) posterior end of a *Procyrnea* *leptoptera* adult male, scale bar 350 µm.

**Figure 6:**
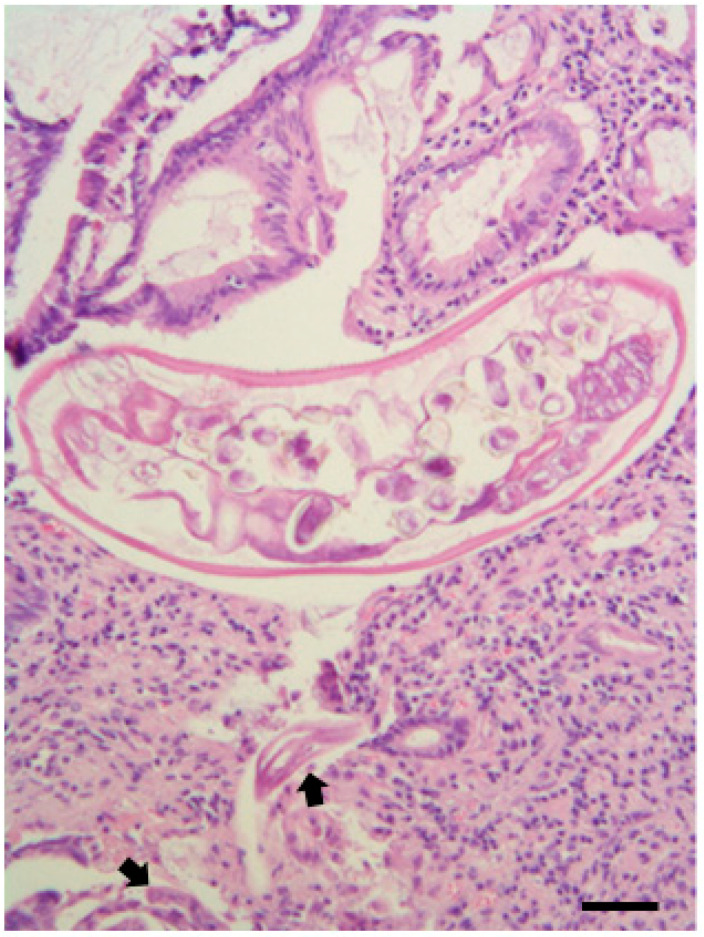
Eurasian sparrowhawk (*A. nisus*), the presence of parasites (probably *Procyrnea* spp.) in the duodenum. Chronic-active inflammation, characterized by the presence of heterophils, lymphocytes, and macrophages infiltrating a segmental tract of the organ wall, is observed. Note the presence of some parasites in the endoluminal area (arrows) not encysted or surrounded by a granulomatous reaction. H&E, scale bar = 150 µm.

**Figure 7:**
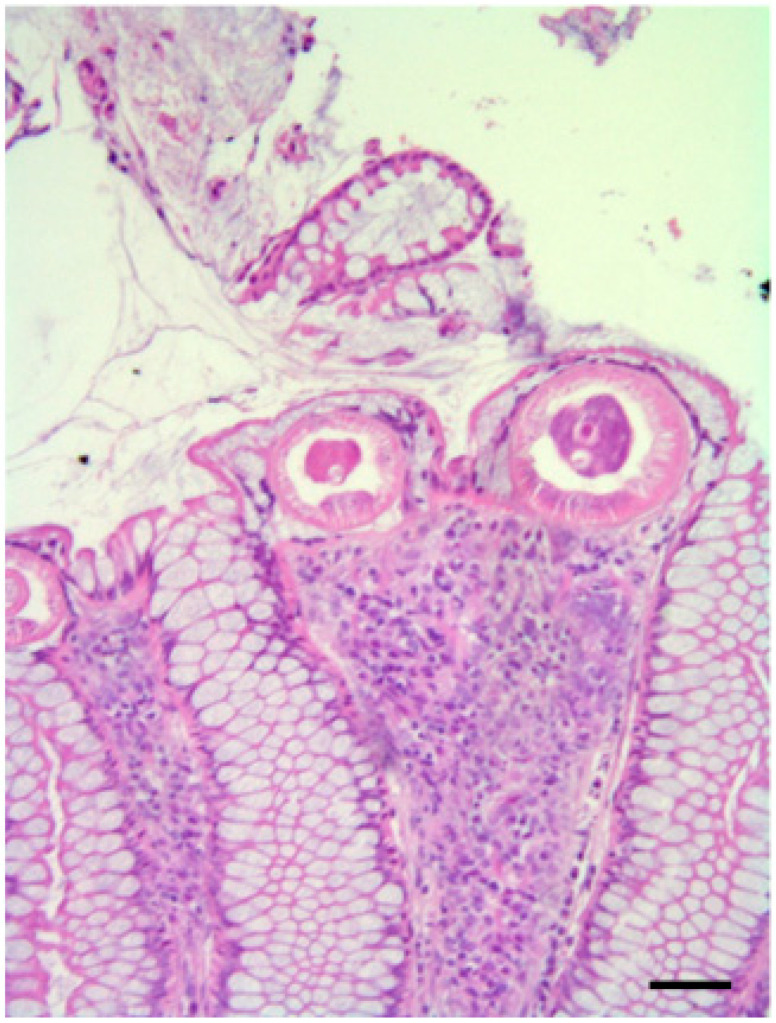
Presence of sub-epithelial parasites in the intestine of a *B. falconis*-infected Eurasian sparrow hawk (*A. nisus*). Diffuse chronic interstitial inflammation with the presence of transmural infiltrate of lymphocytes and plasma cells in the presence of parasites. Note the presence of diffuse mucinous metaplasia of the colonic mucosa, with a total transformation due to an overgrowth of goblet cells. H&E; scale bar = 150 µm.

Among positive sparrowhawks, sparrowhawk 1 showed catarrhal enteritis at necropsy, but no pathogens were detected at the bacteriological examination (Table 1 and Table 4). However, at the parasitological analysis, *P. leptoptera*, *Synhimantus* (*Dispharynx*) *falconis,* and *P. alata* were identified in the proventriculus and the proximal tract of the small intestine (Table 2, Figure 5). At the histopathological examination, these parasites were found associated with foci of chronic-active inflammation, characterized by the presence of heterophiles, lymphocytes, and macrophages infiltrating the proventriculus and a segmental tract of the duodenal wall; parasites were also evidenced in the lumen of these organs (Table 3, Figure 6). As observed in the common buzzards, *B. falconis* found in the small intestine of this animal was instead associated with foci of acute inflammation of jejunum and ileum and with the presence of micro-agglomerates of heterophils and eosinophils, areas of mucosal erosion, and hyperemia (Table 3, Figure 7).

Parasitological analysis of sparrowhawk 2 (Table 2) revealed *P. leptoptera* infection in the gizzard and the proventriculus associated with moderate histopathological lesions characterized by micro- and macro-granulomatous foci and ulcers on the mucosa overlying the micro-granulomatous areas (Table 3). Granulomas were characterized by a necrotic center with the presence of dystrophic calcification, with the cell wall consisting of numerous heterophilic granulocytes and macrophages and sometimes of giant cells and mixed lymphocytes and/or plasma cells. These findings agree with previous observations in sparrowhawks infected by *P. leptoptera* and with findings from this study in *P. leptoptera* and *P. mansioni*-infected honey and common buzzards, respectively [30,33].

Moreover, *B. falconis* and *Spirocerca* cysts (Table 2), each containing an encysted larva, were identified in the intestine of this animal. At the histopathological examination, capillariids were evidenced in the lumen and within the mucosa of the intestine associated with diffuse chronic interstitial inflammation with the presence of transmural infiltrate of lymphocytes and plasma cells (Table 3, Figure 7).

Microscopic examination of the intestinal content of this animal also revealed the presence of a high number of protozoan sporocysts, with a mean measurement of 12.35 × 8.72 µm (range 10.9–13.08 × 8.72 µm) (Table 2), morphologically identifiable with *Sarcocystis columbae* [59]. In the whole intestinal tract, lesions and chronic diffuse inflammation were highlighted at the histological examination, with the presence of transmural infiltrates of lymphocytes and plasma cells associated with a high number of endocellular protozoa in the epithelium and the lamina propria of the small intestine (Table 3), concurrent with evident bacterial overgrowth in the crypts and glands of the intestinal mucosa.

At necropsy (Table 1), this animal also showed necrotic enteritis, and *Clostridium perfringens* intestinal infection was identified at bacteriological analysis (Table 4).

*C. perfringens* is often a component of the normal intestinal microbiome of various birds, and it is particularly frequent in Falcon raptors [18,60]. However, in the presence of concurrent factors, such as diet changes, stressful conditions, and some intercurrent infections, it can cause necrotic enteritis with diarrhea, often bloody, progressive dehydration that may also lead diseased birds to death, and secondary hepatitis and splenitis [61,62]. In poultry, *C. perfringens* is the most important causative agent of necrotic enteritis [63,64], and this condition is especially observed when bacterial overgrowth concurs with a severely damaged intestinal epithelium, often due to heavy coccidian infections [64]. Therefore, the concurrent severe *S. columbae* infection and necrotic enteritis may suggest a possible role of this protozoan infection as a predisposing factor for the onset of clostridial necrotic enteritis in this bird.

Finally, sparrowhawk 3 showed hemorrhagic gastroenteritis at necropsy (Table 1). The bacteriological analysis was negative, while *B. falconis* and *P. angusticolle* were evidenced in the intestinal tract at the parasitological examination (Table 2, Figure 5). The histopathological examination confirmed the presence of these nematodes in the endoluminal area or attached to the mucosa, and associated lesions were similar to those observed in the *B. falconis*-infected sparrowhawk 2 (Table 3).

At necropsy (Table 1), kestrel 1 showed serous and fibrin collection in the thoracoabdominal cavity and catarrhal-hemorrhagic enteritis, while the liver was dark reddish-brown in color. Three parasite species (Table 2), namely *Diplotriaena falconis*, *Neodiplostomum spathoides,* and *Spirocerca* spp., were identified in the intestine of this animal.

At bacteriological analysis (Table 4), *Yersinia enterocolitica* and *P. multocida* were identified in the intestine and in the brain. Y. *enterocolitica* has been reported in wild-living birds [65,66], including raptors [67]. Infected birds may die very rapidly after infection, or the disease may take weeks to manifest [68]. Intestinal mucinosis associate with *Y. enterocolitica* infection has been also reported [69]. On the other hand, *P. multocida* is the etiological agent of avian cholera and one of the bacterial species more frequently causing disease in birds [46,70]. Severe hyperacute and acute infections may occur, while hemorrhagic diarrhea, along with other signs, can be observed in sub-acute infections [46,70]. Chronic forms include serositis, necrotic foci, and small granulomatous lesions affecting all organs.

**Figure 8:**
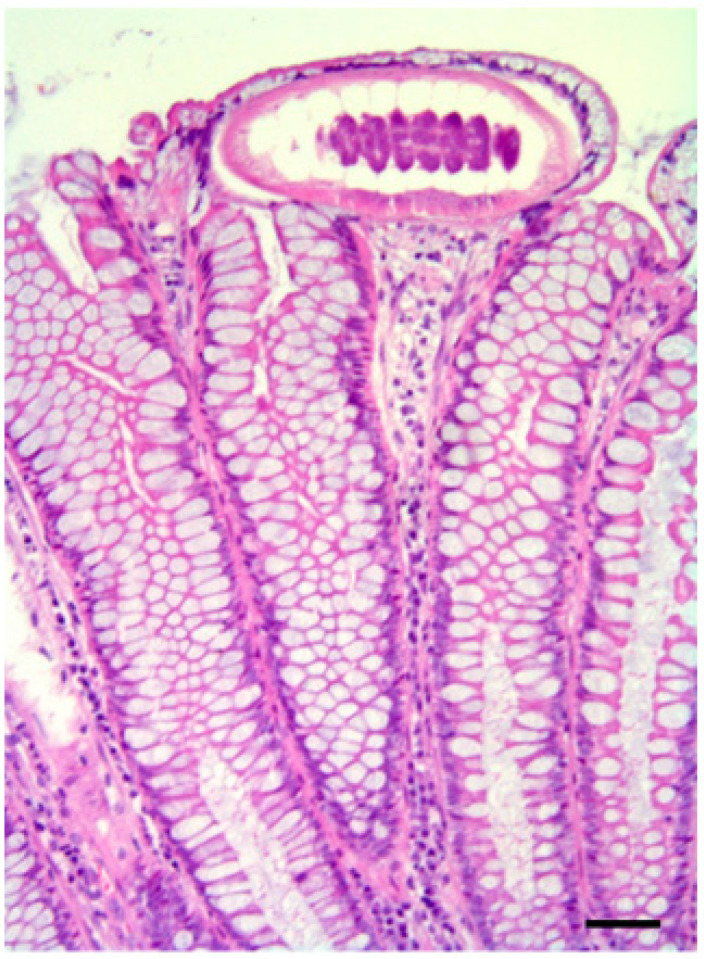
Large intestinal mucinosis and sub-epithelial endowed parasites in a *Spirocerca* spp. infected common kestrel (*F. tinnunculus*). Note the low chronic inflammatory infiltrate, constituted by scattered interstitial lymphocytes, without granulomatous reactions. H&E; scale bar = 150 µm.

Several intestinal macro-granulomatous foci and intestinal mucinosis were observed at the histopathological examination in this bird. Interestingly, a weak inflammatory infiltrate, associated with the areas of colonic mucinosis, was typically associated to the sub-epithelial localization of some *Spirocerca* spp. larvae (Figure 8).

However, serositis observed in the thoracoabdominal cavity, catarrhal-hemorrhagic enteritis, and intestinal macro-granulomatous lesions, as well as liver pathological changes observed in this bird, could be due mainly to *P. multocida*, while *Y. enterocolitica* infection should be considered co-responsible for enteritis and sepsis and co-responsible or the main cause for intestinal mucinosis observed in this bird.

**Figure 9:**
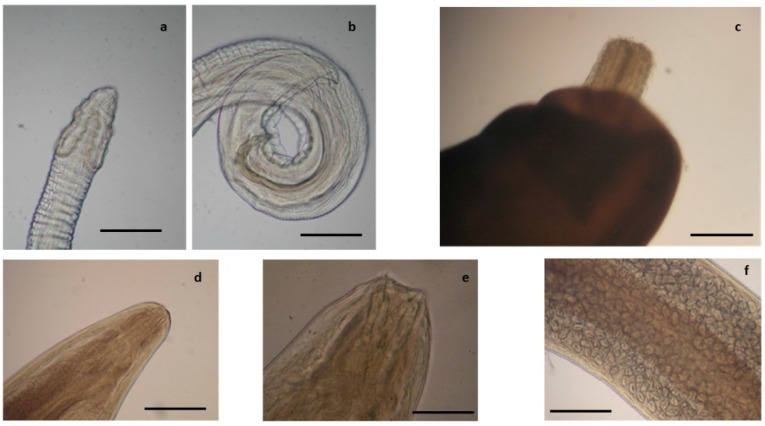
Some of the helminth species identified in the common kestrel (*F. tinnunculus*). Anterior (**a**) and posterior (**b**) end of *Synhimantus laticeps* adult male, scale bar 300 µm. (**c**) Anterior section of the body of *Centrorhynchus falconis* (**d**), scale bar 0.5 mm Anterior end of *Diplotriaena falconis,* scale bar 250 µm. Details of the body of *D. falconis*: (**e**) anterior end and (**f**) section of the body of a female specimen filled with eggs, scale bar 250 µm.

The common kestrel 2 did not show any significant lesions at necropsy, and no bacterial pathogens were identified (Table 1 and Table 4). On the other hand, over 50 *Synhimantus laticeps* adults and a *C. falconis* acanthocephalan were found in the proventriculus and the intestine, respectively (Table 2, Figure 9). The histopathological examination revealed erosions of the stomach mucosa and the presence of parasites, some of which calcified, in the muscle fibers of the stomach wall that can be associated with the heavy *S. laticeps* infection observed in this bird, as previously reported [30,33].

Finally, among diurnal raptors, the single osprey examined was found positive for *Procyrnea leptoptera* nematoda in the gizzard and proventriculus and for the acanthocephalan species *Andracantha mergi* in the intestine, but no bacteria were found.

Regarding nocturnal raptors, the two scops owls scored negative at the parasitological and bacteriological examinations.

**Figure 10:**
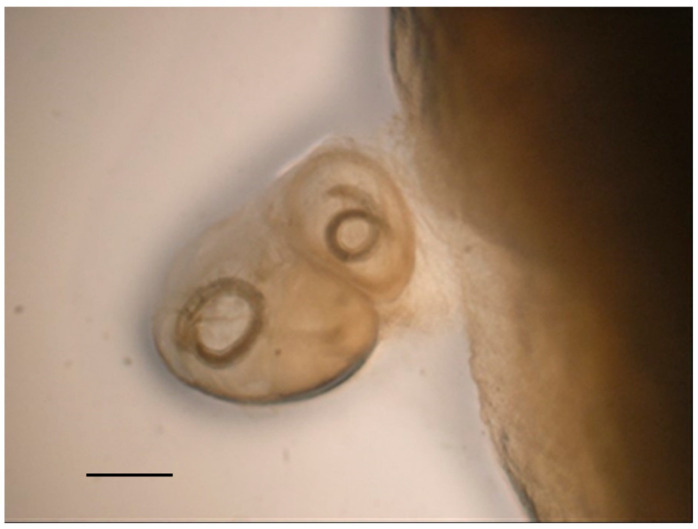
*Spirocerca* spp. cysts in the intestinal wall of the barn owl 2, scale bar 750 µm.

The barn owl 1 (Table 1) showed enteritis associated with *Sarcocystis dispersa* infection, but no bacteria were identified, while parasitological and histopathological examinations of the barn owl 2 revealed *Spirocerca* spp. cysts in the gastric and intestinal wall along with enteritis and macro-granulomatous lesions (Table 1,Table 2 and Table 3; Figure 10).

**Figure 11:**
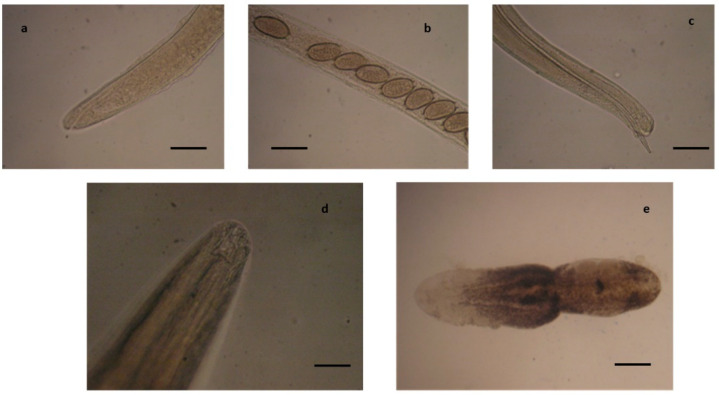
Helminths identified in the little owls. Anterior end (**a**) and a detail of the body with eggs (**b**) of an adult female and caudal end (**c**) of an adult male of *Capillaria tenuissima,* scale bar 80 µm. (**d**) Anterior end of *Procyrnea leptoptera,* scale bar 350 µm. (**e**) *Neodiplostomum attenuatum,* scale bar 350 µm.

Among positive little owls, the parasitological examination (Table 2, Figure 11) revealed *Capillaria tenuissima* infection in the small intestine of almost all of these birds, while little owl 2 was also found infected by *P. leptoptera* in the gizzard and proventriculus, and little owl 12 was infected by *N. attenuatum* in the intestine (Table 2). Bacteriological analysis revealed *E. coli* infection in the liver of little owl 6 and *P. multocida* infection in the liver, brain, and other organs of little owl 13, showing intestinal gross lesions, hepatomegaly, and obstruction of the cloaca, suggesting septicemia (Table 4). At the histopathological examination (Table 3), diffuse chronic inflammation with transmural infiltrates of lymphocytes and plasma cells was instead highlighted in the gizzard, proventriculus, and the entire intestinal tract of this bird, which was probably caused by *P. multocida* [46,70]. Severe intestinal dysmicrobism and damage to the intestinal epithelium, especially in the duodenum, were also evidenced.

**Figure 12:**
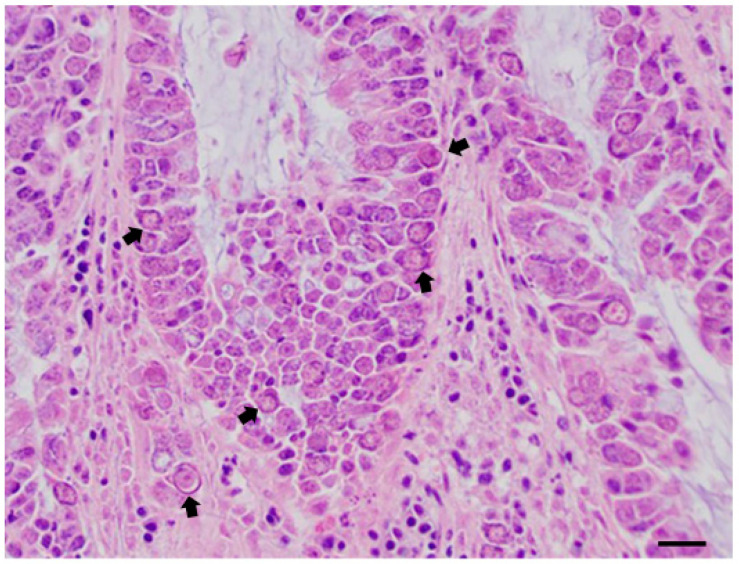
Jejunal mucosa of little owl (*A. noctua*); chronic inflammatory infiltrate in an interstitial diffuse form, associated with the large presence of apicomplexan protozoa in the epithelium (various stages of the life cycle—arrows) or inside macrophages interspersed in the intestinal wall. H&E; scale bar = 100 µm.

*C. tenuissima* infection was instead found constantly associated with diffuse chronic inflammation of mild severity with transmural infiltrates of lymphocytes and plasma cells or with foci of acute inflammation, micro-aggregates of heterophils, and eosinophils. Areas of mucosal erosion and hyperemia were instead found associated with *N. attenuatum* infections. Moreover, mild atypical micro-granulomatous lesions were also found in the jejunal and ileal tract of little owl 6, probably due to *E. coli* infection [18]. In little owl 12, a heavy colonization of coccidian parasites, identifiable with *Eumonospora mochogalegoi* or *Eumonospora henryae* previously reported in the little owl [71], was also evidenced at histopathology (Figure 12).

## 3. Materials and Methods

### 3.1. Animals

Twenty-nine deceased diurnal and nocturnal raptors were examined from 2003 to 2005 for endoparasites and bacteria (Table 1). The examined diurnal raptors (Accipitriformes and Falconiformes) included four common buzzards (*B. buteo*), two European honey buzzards (*P. apivorus*), three sparrowhawks (*A. nisus*), two common kestrels (*F. tinnunculus*), and a western osprey (*P. haliaetus*) (Table 1). Among nocturnal raptors (Strigiformes), two barn owls (*T. alba*), thirteen little owls (*A. noctua*), and two scops owls (*O. scops*) were examined (Table 1). All the raptors examined in this study had died from a few hours to ten days after their arrival in wildlife rescue centers located in the district of Lucca (43°50′ N, 10°30′ E) (Tuscany, central Italy), with the exception of a barn owl (*T. alba*) that was found dead in a wildlife rescue center located in the district of Livorno (43°33′ N, 10°18′ E, Tuscany, central Italy) and a common kestrel (*F. tinnunculus*) that was found dead in the district of Pisa (43°43′ N, 10°23′ E, Tuscany, central Italy). The examined birds had not been treated with antiparasitic drugs and were single-caged. In most cases, deceased birds were placed in clean bags, refrigerated, and transported to the laboratory soon after their death. All examined birds were necropsied, and bacteriological and parasitological analysis of the different tracts of the digestive system were performed. Organs that scored positive for the presence of endoparasites and/or bacteria underwent further histopathological examinations.

### 3.2. Necropsy

An external examination was performed to evaluate eventual abnormalities of muscle masses, subcutaneous fat, and mucous membranes. All organs and systems were examined to assess the presence of macroscopic lesions.

### 3.3. Bacteriological Analysis

Brain, intestine, and liver samples taken from all birds were cultured on several culture media specific for the search of different selected bacteria, including *Salmonella* spp., *Escherichia coli*, *Yersinia* spp., *Pasteurella* spp., and *Clostridium* spp. [72].

For *Salmonella* spp., samples were inoculated in Buffered Peptone Water and incubated at 37 °C for 18 h. After incubation, the samples were inoculated into Rappaport-Vassiliadis soya broth (Oxoid, Milan, Italy) and incubated at 42 °C for 18 h. The cultures obtained were plated onto Xylose-lysinedeoxycholate agar (Oxoid, Milan, Italy) and Brilliant Green Agar (Oxoid, Milan, Italy), incubated at 37 °C, and examined after 24 h. Suspected colonies were then sub-cultured in nutrient agar, and the confirmation of *Salmonella* species was performed using oxidase, API 20E (BioMérieux, Florence, Italy), and polyvalent antisera. *Salmonella* spp. isolates were serotyped according to the Kauffmann–White scheme in collaboration with The Reference Center for Pathogenic Enterobacteria (IZSLT, Rome, Italy).

To isolate *E. coli*, samples were inoculated onto Colombia blood agar base (Oxoid, Milan, Italy) and MacConkey agar (Oxoid, Milan, Italy) and incubated at 37 °C for 18 h. The biochemical identification was performed using a miniaturized biochemical test galleries API 20E system (BioMérieux, Florence, Italy).

For *Clostridium perfringens* isolation, samples were inoculated onto Colombia blood agar base (Oxoid, Milan, Italy), incubated at 37 °C for 24–48 h anaerobically, and the glove box was used with the AnaeroGen (Oxoid, Milan, Italy). The identification of colonies with specific characteristics (β-haemolytic and lecithinase positive) was performed using API rapid 32A and API 20A (BioMérieux, Florence, Italy).

The isolation of *Yersinia* spp. was performed by direct inoculation in cold pre-enrichment broth phosphate-buffered saline and incubated at 25 °C for 48 h. After incubation, the samples were inoculated onto Colombia blood agar base (Oxoid, Milan, Italy), MacConkey agar (Oxoid, Milan, Italy) and *Yersinia* selective agar base cefsulodin-irgasan-novobiocin (Oxoid, Milan, Italy) with incubation at 30 °C for 24–48 h. The confirmation of *Yersinia enterocolitica* was performed using oxidase, urease, and miniaturized biochemical test by using test galleries API 20E (BioMérieux, Florence, Italy).

For *Pasteurella multocida,* samples were inoculated onto Colombia blood agar base (Oxoid, Milan, Italy), MacConkey agar (Oxoid, Milan, Italy), and brain heart infusion agar broth and incubated at 24–48 h at 37 °C. The confirmation of *P. multocida* was obtained using oxidase and miniaturized biochemical test galleries API 20 NE (BioMérieux, Florence, Italy).

### 3.4. Parasitological Analysis

The whole digestive (liver, gall bladder, esophagus, stomach, duodenum, jejunum-ileum, ceca, and cloaca) system was opened. A batch of intestinal tract content samples was microscopically examined under an optical microscope, both as fresh smears and after the flotation test with saturated NaCl solution (specific gravity 1.2), to detect the presence of nematode eggs or protozoa cysts/oocysts/sporocysts. Then, each opened digestive tract was washed in saline and subjected to sedimentation in a refrigerator for approximately 24 h. After this time, the sediment was observed under a stereoscope to assess the presence of adult helminths that were collected, counted, washed in saline solution, and fixed in a glycerin-ethanol solution (medium of Looss). Helminths were cleared in lactophenol on a glass slide for identification under an optical microscope and then returned to the preservative. All measurements were taken with the aid of a micrometric eyepiece.

The identification of parasite genus/species was performed based on keys or descriptions given in previous studies [40,41,48,59,73,74,75,76,77,78,79,80,81,82,83,84].

### 3.5. Histopathological Analysis

Organs from 18 raptors, including common buzzards (*B. buteo*, *n* = 4), Eurasian sparrowhawks (*A. nisus*, *n* = 3), European honey buzzards (*P. apivorus*, *n* = 2), common kestrels (*F. tinnunculus*, *n* = 2), barn owls (*T. alba*, *n* = 1), and little owls (*A. noctua*, *n* = 6), scored positive for parasites and/or bacteria, were processed for histopathological examination.

Fragments of at least 2.5 cm in length of the esophagus, proventriculus, ventriculus, duodenum, jejunum/ileum, and colon were collected. Samples were fixed in 10% neutral buffered formalin for a period of 24 h and routinely processed. Three-micrometer paraffin sections were placed on Superfrost Plus slides (Histoline, Milan, Italy). The slides were then dewaxed and stained with hematoxylin and eosin stain (H&E) for microscopic examination. The morphological evaluation of each digestive tract was carried out, with the determination of the location of the parasite and, in the case of an evident histological lesion, the characterization of the inflammation (acute, chronic-active, granulomatous inflammation). Moreover, the sections found infected by parasites were stained with Shiff’s Periodic Acid (PAS).

The following parameters were used for the histological evaluation: for each lesion, a score evaluation was performed considering as: grade 0 (negative = lesion not observed); grade from 1 to 3 (indicated with the + sign) the progressive degree of the lesions. The lesion score was therefore 1 (+) = minor lesion; 2 (++) = moderate lesion; 3 (+++) = severe lesion. To characterize the lesions, a letter from A to G was used, as described in the caption of Table 3.

## 4. Conclusions

Worldwide, raptors are included among animals more frequently admitted to wildlife recovery centers. Moreover, many raptor populations are highly threatened, mainly due to human activities. Therefore, the collection of data on pathogens and associated lesions in raptors living in a specific area can provide useful information for enhancing raptor conservation efforts, reducing pathogen transmission between raptors, domesticated animals, and humans, and improving the knowledge on raptor pathogens.

To the best of the authors’ knowledge, this study is the first organic investigation on endoparasite infections and associated bacteria and pathological lesions of the digestive tract of European raptors. Although most of the pathogens identified in this study are commonly found in European diurnal and nocturnal raptors, this study is the first report of *S. typhimurium* and *S. enterica* subspecies *diarizonae* in the common buzzard (*B. buteo*) and the first report in Italy of the parasite species *Andracantha mergi*, *Spirocerca* spp., *Sarcocystis dispersa*, *Sarcocystis columbae,* and *Eumonospora* spp.

The results obtained showed that both parasites, especially when in high number, and/or pathogenic bacteria may cause severe lesions in the digestive system of diurnal and nocturnal raptors; parasites and bacteria may concur in causing these lesions; and most severe lesions are caused by the interaction of multiple pathogens, both parasites and bacteria. Moreover, in most cases, the same parasite genus/species was frequently found associated with the same type of lesions in the histopathological analysis. The potential role of these birds for the dispersal of bacteria potentially pathogenic for humans, such as *Salmonella* spp. and *Y. enterocolitica*, and domesticated animals was also highlighted. Although the characterization of some bacteria, such as *E. coli*, was not performed in this study, the obtained data are indicative of associated septicemic forms. Finally, the severity of pathological changes found associated with parasitic and/or bacterial pathogens in some of these raptors could have greatly impaired the function of the digestive system and, in some cases, may have represented the main cause for the death of these birds.

## Figures and Tables

**Table 1:** Examined diurnal (12) and nocturnal (17) raptors deceased in wildlife rescue centers in Tuscany (central Italy) and the results of post-mortem gross examination.

	Common Name and Identification Number	Species	Sex	Age (Young/Adult)	Necropsy (*post-mortem* Gross Examination)
	**Diurnal Raptors**				
1	Osprey 1	*Pandion haliaetus*	Male	Young	Reduced muscle mass and enteritis
2	Common buzzard 1	*Buteo buteo*	Female	Adult	Left ulna fracture due to a gunshot and cranial hematoma
3	Common buzzard 2	*B. buteo*	Female	Adult	Dehydration and reduced muscle mass; cloaca, left oviduct, and right ventricle enlarged; congestion of the gastric and small intestine mucosa
4	Common buzzard 3	*B. buteo*	Male	Young	Severe anemia; reduced and pale muscle masses; head trauma; hypertrophic heart; the presence of mucus in the esophagus
5	Common buzzard 4	*B. buteo*	Male	Young	Gunshot wound with an open fracture of the right humerus; reduced muscle mass; necrotic esophageal fistulous lesions; thickened thoracic air sacs and pneumonia
6	Honey buzzard 1	*Pernis apivorus*	Male	Young	Reduced muscle mass; necrotic esophageal lesions
7	Honey buzzard 2	*P. apivorus*	Male	Young	Severe weight loss and dehydration; mild catarrhal enteritis; hepatomegaly and nephromegaly
8	Sparrowhawk 1	*Accipiter nisus*	Male	Young	Severe cachexia and catarrhal enteritis
9	Sparrowhawk 2	*A. nisus*	Female	Adult	Fracture of the left humerus for a gunshot; necrotic enteritis; and the presence of digested blood in the stomach and in the intestine
10	Sparrowhawk 3	*A. nisus*	Female	Adult	Right paw dermatitis; old fracture of the humerus; hemorrhagic gastroenteritis and fibrin deposits in the air sacs; liver dark red in color
11	Common kestrel 1	*Falco tinnunculus*	Female	Adult	Fracture of the right tarsometatarsal joint; serous collection in the thoracoabdominal cavity and catarrhal-hemorrhagic enteritis
12	Common kestrel 2	*F. tinnunculus*	Female	Adult	Reduced muscle mass
	**Nocturnal Raptors**				
13	Barn owl 1	*Tyto alba*	-	Adult	Enteritis
14	Barn owl 2	*T. alba*	Male	Adult	Cachexia and hemorrhagic and catarrhal enteritis
15	Little owl 1	*Athene noctua*	Female	Adult	Congested kidneys
16	Little owl 2	*A. noctua*	Female	Young	Reduced muscle mass
17	Little owl 3	*A. noctua*	Male	Young	Very young subject, still not fully able to fly
18	Little owl 4	*A. noctua*	Male	Young	Very young subject, still not fully able to fly
19	Little owl 5	*A. noctua*	Male	Young	Very young subject, still not fully able to fly; reduced muscle mass
20	Little owl 6	*A. noctua*	Male	Young	Mild congestion of the mucous membrane of the first intestinal tract, cloacal obstruction
21	Little owl 7	*A. noctua*	Female	Young	Very young subject, still not fully able to fly
22	Little owl 8	*A. noctua*	Male	Young	Very young subject, still not fully able to fly
23	Little owl 9	*A. noctua*	Male	Young	Cachexia.
24	Little owl 10	*A. noctua*	Male	Young	Very young subject, still not fully able to fly
25	Little owl 11	*A. noctua*	Female	Adult	Spinal cord hematoma
26	Little owl 12	*A. noctua*	Female	Adult	Gunshot wound in the pectoral muscles and on the right side of the neck; enteritis, lungs congested and hemorrhagic
27	Little owl 13	*A. noctua*	Female	Adult	Cachexia, nephritis, and pulmonary congestion
28	Scops owl 1	*Otus scops*	Male	Adult	Gunshot wound to the right wing; reduced muscle mass and pyoderma in the sternal region
29	Scops owl 2	*O. scops*	Male	Young	Enlarged stomach with catarrhal-hemorrhagic gastroenteritis and necrotic areas in the liver

**Table 2:** Endoparasite species (Phylum: Family) identified in the digestive system of examined raptors.

Animal	Infected Organs	Nematodes	Cestodes	Trematodes	Acanthocephala	Protozoa
Osprey 1	Gizzard, Proventriculus, Intestine	*Procyrnea leptoptera* (Nematoda: Habronematidae) 3 *	-	-	*Andracantha mergi* (Acanthocephala: Polymorphidae) 2 *	-
Common buzzard 1	Gizzard, Proventriculus, Intestine	*Physaloptera alata* 1 * (Nematoda: Physalopteridae) *Porrocaecum angusticolle* (Nematoda: Ascarididae) 1 * Baruscacapillaria falconis (Nematoda: Capillariidae) 3 * Procyrnea mansioni (Nematoda: Habronematidae) 7 *	-	-	*Centrorhynchus globocaudatus* (Acanthocephala: Centrorhynchidae) 80 *	-
Common buzzard 2	Gizzard, Proventriculus, Intestine	*Procyrnea mansioni* (Nematoda: Habronematidae) 1 * Porrocaecum angusticolle (Nematoda: Ascarididae) 1 * Baruscacapillaria falconis (Nematoda: Capillariidae) 2 * Spirocerca spp. encysted larvae (Nematoda: Thelaziidae) 4 *	-	*Neodiplostomum attenuatum* (Platyhel-minthes: Diplostomatidae) 2 *	*Centrorhynchus globocaudatus* (Acanthocephala: Centrorhynchidae) 2 *	-
Common buzzard 3	Esophagous, Gizzard, Proventriculus, Intestine	*Eucoleus dispar* (Nematoda: Capillariidae) 4 * Porrocaecum angusticolle (Nematoda: Ascarididae) 6 * Baruscacapillaria falconis (Nematoda: Capillariidae) 13 * Procyrnea mansioni (Nematoda: Habronematidae) 50 *	-	*Neodiplostomum attenuatum* (Platyhel-minthes: Diplostomatidae) 50 *	*Centrorhynchus aluconis* (Acanthocephala: Centrorhynchidae) 9 *	-
Common buzzard 4	Esophagous, Gizzard, Proventriculus, Intestine	*Eucoleus dispar* (Nematoda: Capillariidae) 5 * Porrocaecum angusticolle (Nematoda: Ascarididae) 1 * Baruscacapillaria falconis (Nematoda: Capillariidae) 3 * Procyrnea mansioni (Nematoda: Habronematidae) 2 *	*Cladotaenia globifera* (Platyhelminthes: Paruterinidae) 9 *	-	*Centrorhynchus aluconis* (Acanthocephala: Centrorhynchidae) 20 *	-
Honey buzzard 1	Esophagous, Gizzard, Intestine	*Eucoleus dispar* (Nematoda: Capillariidae) 1 * Physaloptera apivori (Nematoda: Physalopteridae) 4 *	*Raillietina apivori* (Platyhelminthes: Davaineidae) 1 *	-	-	-
Honey buzzard 2	Esophagous, Gizzard, Proventriculus, Intestine	*Eucoleus dispar* (Nematoda: Capillariidae) 4 * Procyrnea leptoptera (Nematoda: Habronematidae) 7 * Baruscacapillaria falconis (Nematoda: Capillariidae) 2 *	-	-	-	-
Sparrowhawk 1	Gizzard, Proventriculus, Intestine	*Procyrnea leptoptera* (Nematoda: Habronematidae) 7 * Physaloptera alata (Nematoda: Physalopteridae) 1 * Synhimantus laticeps (Nematoda: Acuariidae) 2 * Baruscacapillaria falconis (Nematoda: Capillariidae) 7 *	-	-	-	-
Sparrowhawk 2	Gizzard, Proventriculus, Intestine	*Baruscacapillaria falconis* (Nematoda: Capillariidae) 3 * Procyrnea leptoptera (Nematoda: Habronematidae) 2 * Spirocerca spp. encysted larvae (Nematoda: Thelaziidae) 3 *	-	-	-	*Sarcocystis columbae* (Apicomplexa: Sarcocystidae) heavy infections
Sparrowhawk 3	Intestine	*Baruscacapillaria falconis* (Nematoda: Capillariidae) 9 * Porrocaecum angusticolle (Nematoda: Ascarididae) 3 *	-	-	-	-
Common kestrel 1	Intestine	*Spirocerca* spp. (Nematoda: Thelaziidae) 2 * Diplotriaena falconis (Nematoda, Diplotriaenidae) 6 *	-	*Neodiplostomum spathoides* (Platyhel-minthes: Diplostomatidae) 1 *	-	-
Common kestrel 2	Intestine, Gizzard, Body Cavity	*Synhimantus laticeps* (Nematoda: Acuariidae) 50 *	-	-	*Centrorhynchus falconis* (Acanthocephala: Centrorhynchidae) 1 *	-
Barn owl 1	Intestine					*Sarcocystis dispersa* (Apicomplexa: Sarcocystidae)
Barn owl 2	Intestine	*Spirocerca* spp. (Nematoda: Thelaziidae) 18 *	-	-	-	-
Little owl 2	Gizzard, Proventriculus, Intestine	*Procyrnea leptoptera* (Nematoda: Habronematidae) 1 * Capillaria tenuissima (Nematoda: Capillariidae) 10 *	-	-	-	-
Little owl 4	Intestine	*Capillaria tenuissima* (Nematoda: Capillariidae) 3 *	-	-	-	-
Little owl 5	Intestine	*Capillaria tenuissima* (Nematoda: Capillariidae) 4 *	-	-	-	-
Little owl 6	Intestine	*Capillaria tenuissima* (Nematoda: Capillariidae) 5 *	-	-	-	-
Little owl 8	Gizzard, Proventriculus	*Procyrnea leptoptera* (Nematoda: Habronematidae) 1 *	-	-	-	-
Little owl 12	Intestine	*Capillaria tenuissima* (Nematoda: Capillariidae) 4 *	-	*Neodiplostomum attenuatum* (Platyhel-minthes: Diplostomatidae) 10 *	*-*	*Eumonosporamochogalegoi/Eumonospora henryae* (Apicomplexa: Sarcocystidae)
Little owl 13	Intestine	*Capillaria tenuissima* (Nematoda: Capillariidae) 2 * Spirocerca spp. (Nematoda: Thelaziidae) 3 *	Unidentified cestode eggs	-	-	-

* Number of parasite specimens counted.

**Table 3:** Results of histopathological analysis of the different digestive system tracts of the 18 examined raptors. Score lesion is: 0 (lesion not observed); 1(+) = mild injury; 2(++) = moderate injury; 3(+++) = severe injury. NS = organ not sampled.

Raptor Species	Esophagus	Proventriculus	Gizzard	Duodenum	Jejunum/Ileum	Colon
Common buzzard (*B. buteo*) 1	ns	0	D2 +++	0	0	D +++
Common buzzard (*B. buteo*) 2	ns	D1 +++	G +	0	E ++;	D ++; F +
Common buzzard (*B. buteo*) 3	ns	D1++	D2 +	C+++	E ++; F +++	E +; F ++;
Common buzzard (*B. buteo*) 4	C ++	A +++, D1 +	B+, D++	A+++	A +, F +, G++	0
European honey buzzard (*P. apivorus*) 1	F +	G +	0	0	F +	E ++; F +++
European honey buzzard (*P. apivorus*) 2	D+	D1 ++	D2++	C+++	B; C +	C++
Eurasian sparrowhawk (*A. nisus*) 1	ns	ns	ns	B ++	A+	0
Eurasian sparrowhawk (*A. nisus*) 2	ns	D1 ++	D +	C3 +++; F+	C2++; C3+	C2+++; C4 ++
Eurasian sparrowhawk *(A. nisus)* 3	ns	0	0	C3++; F +	F +	0
Common kestrel (*F. tinnunculus*) 1	0	G +	0	0	D ++; F +	C2+++; C4 ++
Common kestrel (*F. tinnunculus*) 2	ns	0	0	0	0	0
Barn owl (*T. alba*) 2	ns	0	D +++	0	D +	0
Little owl (*A. noctua*) 2	ns	0	0	0	A+	0
Little owl (*A. noctua*) 4	ns	C +	0	0	C++	0
Little owl (*A. noctua*) 5	ns	0	0	C+	C+	0
Little owl (*A. noctua*) 6	ns	D +	0	0	E +	0
Little owl (*A. noctua*) 12	ns	D +	0	0	C++; C3 +++	A +++
Little owl (*A. noctua*) 13	ns	C +++	C +	C1 +++	C1 ++	C ++

**A:** Focus of acute inflammation, with the presence of micro-thickened heterophiles and eosinophils, with areas of mucosal de-epithelialization, as well as the presence of hyperemia. Absence of visible parasites; **B**: Focus of chronic-active inflammation, characterized by the presence of heterophiles, lymphocytes, and macrophages infiltrating a segmental tract of the organ wall, with the presence of parasites in the endoluminal area but not encysted or with a granulomatous reaction; **C**: Diffuse chronic inflammation with the presence of transmural infiltrate of lymphocytes and plasma cells in the absence of visible parasites; **C1**: As above, but with an evident dysmicrobism, characterized by a strong presence of Gram-negative/Gram-positive bacteria and damage to the intestinal epithelium; **C2**: As above, but with evident clostridial overgrowth (presence at the level of the crypts and glandular lumens of the mucosa of large quantities of filamentous rod-cell bacteria with clear endosomatic endospore); **C3**: As above, but with the evident presence of endocellular protozoa in the epithelium (various phases of the cycle) or inside macrophages interspersed in the intestinal wall; **C4**: As above, but with the evident presence of sub-epithelial parasites encysted at the apical/superficial level of the intestinal mucosa. **D**: Micro- and macro-granulomatous foci, characterized by the presence of parasites (larvae/adults) in the muscle wall. Granulomas characterized by a necrotic center with the presence of dystrophic calcification, a cell wall consisting of numerous heterophilic granulocytes and numerous macrophages or sometimes of giant cells and mixed lymphocytes and/or plasma cells; **D1**: As above, but with the presence of ulcerations in the mucosa above the micro-granulomatous areas; **D2**: As above, but with lesions located mainly in the isthmus area (anatomical area between the glandular stomach and the muscular stomach); **E**: The presence of lesions of atypical micro-granulomatous type (type “typhoid nodules” = foci of caseous necrosis of sub-miliary size with around some epithelioid cells or 2–3 macrophages and lymphocytes, in the absence of an encapsulating reactive fibrous wall); **F**: The presence of parasites in the endoluminal area or attached to the mucosa, in the absence of specific lesions; **G**: The presence of parasites inside the muscle fibers of the wall, in the absence of pericystic reaction (with few calcified cysts).

**Table 4:** Selected bird bacteria identified in examined raptors.

Animal	Organ	Bacteria
Common buzzard 2	Liver and intestine (and lungs)	*Salmonella typhimurium* (4,5, 12: i: 1,2) Gr O: 4 (B)
Common buzzard 3	Liver and intestine	*Salmonella enterica* subspecies *diarizonae* IIIb (50: r: 1, 5.7) Gr O: 50 (Z)
Honey buzzard 1	Intestine	*Salmonella typhimurium* (4,5, 12: i: 1,2) Gr O: 4 (B)
Honey buzzard 2	Liver (and brain)	*Escherichia coli*
Sparrowhawk 2	Intestine	*Clostridium perfringens*
Common kestrel 1	Intestine (and brain)	*Yersinia enterocolitica* *Pasteurella multocida*
Little owl 6	Liver	*Escherichia coli*
Little owl 13	Liver (and brain)	*Pasteurella multocida*

## Data Availability

Not applicable.
